# Automatic detection of multilayer hexagonal boron nitride in optical images using deep learning-based computer vision

**DOI:** 10.1038/s41598-023-28664-3

**Published:** 2023-01-28

**Authors:** Fereshteh Ramezani, Sheikh Parvez, J. Pierce Fix, Arthur Battaglin, Seamus Whyte, Nicholas J. Borys, Bradley M. Whitaker

**Affiliations:** 1 Electrical and Computer Engineering Department, Montana State University, Bozeman, USA; 2 Department of Physics, Montana State University, Bozeman, USA; 3 Materials Science Program, Montana State University, Bozeman, USA; 4 Optical Technology Center, Montana State University, Bozeman, USA

**Keywords:** Two-dimensional materials, Software

## Abstract

Computer vision algorithms can quickly analyze numerous images and identify useful information with high accuracy. Recently, computer vision has been used to identify 2D materials in microscope images. 2D materials have important fundamental properties allowing for their use in many potential applications, including many in quantum information science and engineering. One such material is hexagonal boron nitride (hBN), an isomorph of graphene with a very indistinguishable layered structure. In order to use these materials for research and product development, the most effective method is mechanical exfoliation where single-layer 2D crystallites must be prepared through an exfoliation procedure and then identified using reflected light optical microscopy. Performing these searches manually is a time-consuming and tedious task. Deploying deep learning-based computer vision algorithms for 2D material search can automate the flake detection task with minimal need for human intervention. In this work, we have implemented a new deep learning pipeline to classify crystallites of hBN based on coarse thickness classifications in reflected-light optical micrographs. We have used DetectoRS as the object detector and trained it on 177 images containing hexagonal boron nitride (hBN) flakes of varying thickness. The trained model achieved a high detection accuracy for the rare category of thin flakes ($<50$ atomic layers thick). Further analysis shows that our proposed pipeline could be generalized to various microscope settings and is robust against changes in color or substrate background.

## Introduction

### Computer vision

Object detection is an important computer vision task that deals with detecting instances of visual objects of certain categories in digital images. The goal of object detection is to develop computational models and techniques that provide one of the most basic pieces of information needed by computer vision applications^1^. In other words, the goal of object detection is to determine whether there are any instances of objects from given categories in an image and, if present, to return the spatial location and extent of each object instance^2^. Object detection supports a wide range of applications, including robot vision, consumer markets, autonomous driving, human computer interaction, content based image retrieval, intelligent video surveillance, and augmented reality^2,3^.

Rapid progressions in deep learning and improvements in device capabilities including computing power, memory capacity, power consumption, image sensor resolution, and optics have improved the performance and cost-effectiveness and assisted the spread of vision-based applications. Compared to traditional computer vision techniques, which have a long trial-and-error process, deep learning enables end-to-end object detectors to achieve greater accuracy in tasks such as image classification, semantic segmentation, object detection and Simultaneous Localization and Mapping^4^.

At present, object detection based on deep learning (DL) frameworks fall into two main categories; two-stage detectors, such as the Region-based Convolutional Neural Network (R-CNN)^5^ and its variants^6–8^, and one-stage detectors, such as You Only Look Once (YOLO)^9^ and its variants^10–13^. On public benchmarks, two-stage detectors typically achieve higher accuracy, whereas one-stage detectors are significantly more time-efficient and more suited to real-time applications^14^.

### Two-dimensional materials

Single and few-layers of two-dimensional (2D) materials provide many opportunities to explore quantum phenomena in systems with appealing features such as strong many-body interactions, pristine interfaces, and strong confinement in a single direction^15–18^. In bulk, 2D materials form van der Waals crystals where strong in-plane bonding within a single layer is complemented by significantly weaker van der Waals bonding between the layers^19^. The anisotropy in bonding strengths combined with a chemical structure where the bonds within a single layer are completely passivated enables individual layers of the 2D material to be isolated, forming chemically and structurally robust sheets of atoms that are atomically thick (i.e., 1–3 atomic layers thick). Although ultrathin, these 2D crystallites can have lateral sizes that are 10–100 μm – 10,000–100,000 $\times$ larger than their thickness.

In single- and few-layer form, many 2D materials exhibit excellent optical, electronic, and/or magnetic phenomena that provide means to prepare, interact, and study quantum states in matter^20–24^. Different Van der Waals materials have been used to demonstrate appealing device applications such as emitting diodes. One such material is hexagonal boron nitride (hBN),an isomorph of graphene with a very indistinguishable layered structure, that holds interesting opto-electrical properties merged with mechanical robustness, thermal stability and chemical inertness^25^. With over 1000 known van der Waals 2D materials that span all functionalities (i.e., insulators, metals, semiconductors, ferromagnets, ferroelectrics, etc.), a prolific parameter space of systems and structure-property relationships for quantum, optoelectronic, and magnetic technologies exists^26–28^. The task of exploring the vast world of 2D materials requires the ability to efficiently and reliable produce single- and few-layer samples of 2D materials^29,30^. However, the most widely-used state-of-the-art method for preparing single- and few-layer samples of 2D materials relies on the manual exfoliation of a large population of smaller crystallites from a bulk crystal. The resulting crystallites have a range of thicknesses, and the entire population must be manually searched using optical reflected light microscopy to identify those that have a desired thickness, which is most often the thickness of a single layer. This labor-intensive method for collecting single- and few-layer samples strongly inhibits rapid discovery and exploration of these material systems.

### Automated 2D material detection

Deep learning computer vision methods identify and analyze numerous images into useful information at a fast rate with a high accuracy. Deploying DL algorithms for 2D material search can automate the flake detection task with minimal need for human intervention. So far, there have been various attempts taken toward flake identification in images^31–38^. However, these methods failed to detect flakes within different microscope settings and image magnification unless the algorithms were retrained using the new settings. In this work, we propose a new detection pipeline which not only makes improvements in detection accuracy, but also is able to detect the rare class of thin flakes ($<50$ atomic layers) with a recall of 0.43 and precision of 0.43. We also applied our proposed pipeline to various microscope settings and showed that the algorithm is robust against changes in color settings and magnification.

## Methods

**Figure 1:**
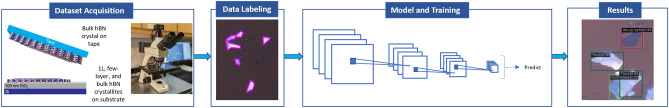
Proposed deep learning pipeline for 2D flake detection. First, a dataset of hBN samples are collected through mechanical exfoliation. Then as the second step, the dataset is annotated manually using an online annotation tool. During the third step, DetectoRS is chosen as the training algorithm, and for the last step, the trained model is applied to the test images to detect hBN samples.

Our proposed deep learning method to detect hBN flakes in 2D material microscopic images is shown as a pipeline in Fig 1. This pipeline consists of three main steps which will be explained in detail.

### Data acquisition

To prepare the dataset, 208 images were collected over 2 months. To do this, hexagonal boron nitride (hBN) samples were fabricated via mechanical exfoliation. Bulk hBN crystals were placed onto a piece of exfoliation tape and separated into thin layers by folding the tape together and peeling the tape apart five times. The exfoliation tape was then placed onto a $1 \times 1$ cm$^2$ silicon (Si) wafer with a 100 nm silicon oxide (SiO$_2$) layer. To ensure the $1 \times 1$ cm$^2$ region of the tape was in contact with the Si/SiO$_2$, the tape was pressed down with a cotton swab across the full area of contact. The sample, Si/SiO$_2$/exfoliation tape, was then heated for 1–2 min at 90 °C and allowed to cool to room temperature, after which the exfoliation tape was removed. The hBN sample images were taken with a 20X objective using a Motic BA310MET-T Incident and Transmitted Metallurgical Microscope. The hBN images for the dataset were taken with the same camera settings and light intensity.

A second dataset of hBN images was collected by our colleagues at the University of Arkansas (UA) following a similar procedure. The UA dataset consists of 10 images that we use exclusively for testing the data. Finally, we use a third dataset for testing purposes: 10 hBN images collected by Masubuchi et al.^32^.

### Data labeling

Labeling the data is the preliminary step in a supervised learning object detection task, and the results of the model heavily depend on the accuracy of the annotations. To annotate our data, we used Roboflow (https://roboflow.com) which is an online annotation tool. For better accuracy of training, we annotated the flakes within each individual image manually and in three different categories as; Thick, Intermediate, and Thin. Table 1 shows the thickness and number of layers for each class. The table also indicates the number of images and the number of labeled instances of each flake type present in the full dataset.

**Table 1:** Dataset Labels and annotations.

Label	Thickness (nm)	Number of atomic layers	Number of annotations		
			Train	Validation	Test
Thick	$\ge$ 50	$\ge$100	996	240	73
Intermediate	25–50	50–100	465	14	12
Thin	$\le$ 25	$\le$50	140	23	7

In addition to annotating our images, we annotated the 10 test images in the UA dataset and re-annotated the 10 test images in the Masubuchi dataset to ensure uniformity in flake labels.

The annotations were then saved in the Microsoft common objects in context (COCO) JSON format. To train and evaluate the machine learning algorithm, the dataset was split into three subsets: training set, validation set and testing set. The training set consists of 177 images which contain 81% of the annotations; the validation set consists of 21 images which contain 14% of the annotations; and the testing set consists 10 images which contain 5% of the annotations.

### Model and training

To detect the 2D objects, DetectoRS^39^ was chosen as the training algorithm. DetectoRS is a multi-stage supervised object detection algorithm. This algorithm involves two major components: Recursive Feature Pyramid (RFP) and Switchable Atrous Convolution (SAC). RFP is employed at the macro level that incorporates extra feedback connections from Feature Pyramid Networks into the bottom-up backbone layers. At the micro level, SAC is utilized, which convolves the features with different atrous rates and gathers the results using switch functions. Combining them results in DetectoRS, which significantly improves the performance of object detection. DetectoRS achieves state-of-the art performance on the COCO test-dev dataset. More information concerning this architecture can be found in the original paper^39^.

The performance of supervised machine learning models depends on the quantity of the dataset to avoid overfitting. However, most of the time collecting and labelling the data is a tedious time-consuming process. Data augmentation techniques are a solution to address this problem. The augmented data will represent a more comprehensive set of possible data points, thus minimizing the distance between the training and validation set, as well as any future testing sets^40^. To augment the data, we used various techniques such as rotating, flipping and color contrast twice; once within Roboflow before exporting the annotation and another time during the training procedure.

To improve the performance of the model, we employed transfer learning. Fine-tuning pre-trained deep networks is a practical way of benefiting from the representation learned on a large database while having relatively few examples to train a model^41^. We took the model which was previously trained on the COCO dataset^42^ and used it as a starting point to retrain it on our own dataset. For training on 2D material images, we chose DetectoRS algorithm with Cascade+ResNet-50 as the backbone detector architecture implemented in MMDetection library^43^. The model was trained with the learning algorithm rate of 0.0025, using Cuda and for 20 epochs.

## Results

**Figure 2:**
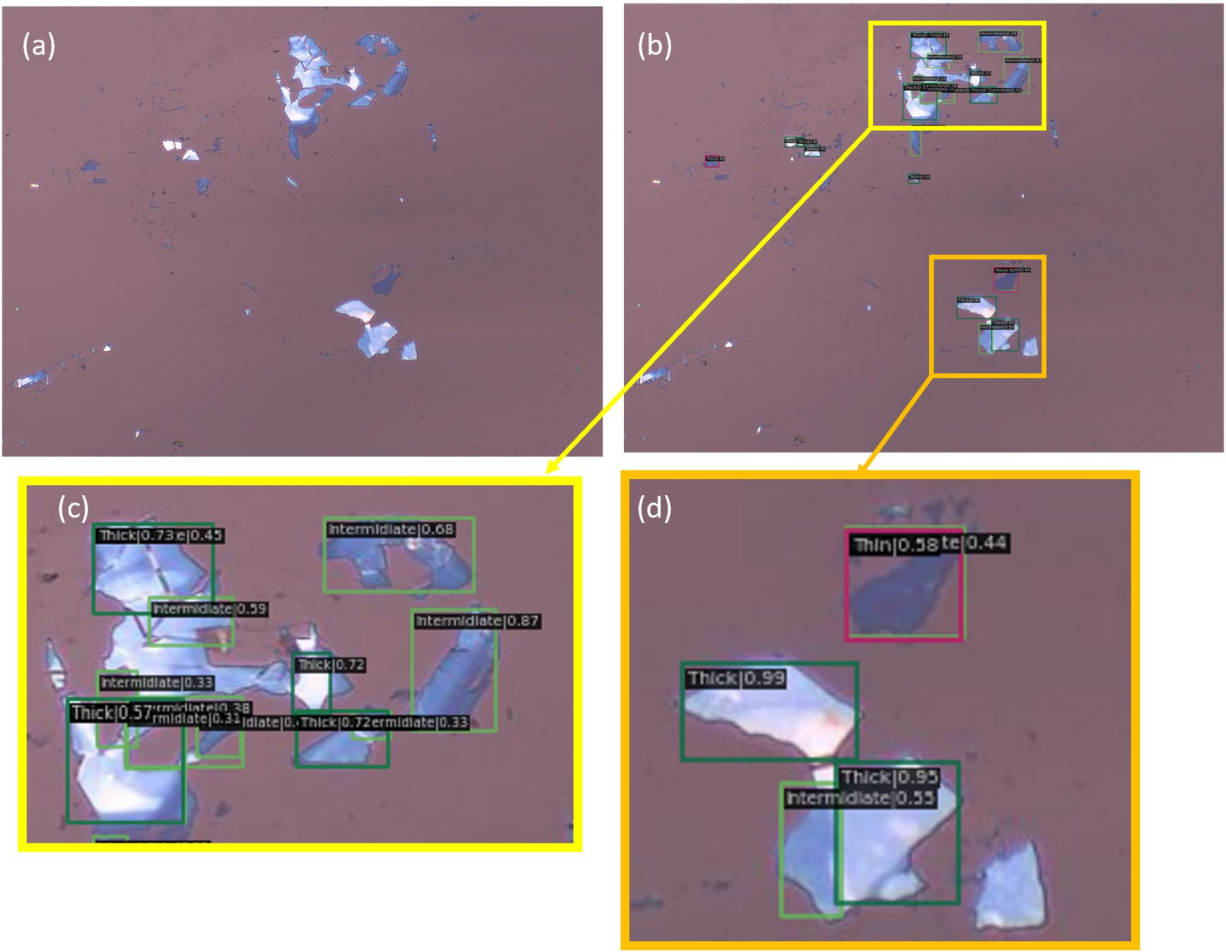
Results obtained from using the proposed algorithm on one image from our dataset; Part (**a**) shows the raw image, part (**b**) shows the labels generated by the algorithm, and parts (**c**) and (**d**) are zoomed-in regions showing the flake type and confidence score reported by the algorithm. We manually identified 19 flakes in the image, and the algorithm correctly classified 14 of them.

The inference results of the trained model when applied to one of the test images is shown in Fig 2. Each flake identification in the image consists of three components; a bounding box, a class label, and a confidence number or probability score which shows the certainty of the algorithm that the class is detected correctly. As can be seen, the algorithm was able to detect the Thin sample, which is the most important among the three classes as it possibly contains a true monolayer material.

To measure the accuracy of the object detector across all test images, we used various evaluation metrics. Before examining these metrics, we present some basic concepts in object detection^44^:

- *True Positive (TP)* A correct detection of a ground-truth bounding box
- *False Positive (FP)* A detection of a nonexistent object or a misplaced detection of an existing object
- *False Negative (FN)* An undetected ground-truth bounding box

A correct or incorrect detection is determined based on Intersection over Union (IoU) which is a threshold. IoU measures how much of a projected object’s bounding box overlaps with the bounding box surrounding the ground reference data.

It is also good to notice that a true negative result is when the algorithm detects the background correctly. Since labeled boundary boxes are only applied to objects, a true negative is not reported in the context of object detection.

Two common metrics to measure the accuracy of the object detection performance are precision and recall which are defined based on the TP, FP and FN: Precision = TP/(TP+FP) and Recall = TP/(TP+FN).

In words, precision returns the ratio of correctly identified objects to all identified objects. Recall measures the proportion of the ground-truth objects that were identified by the algorithm. Mean Average Precision (mAP) is a measure that combines recall and precision for ranked retrieval results^45^.

**Figure 3:**
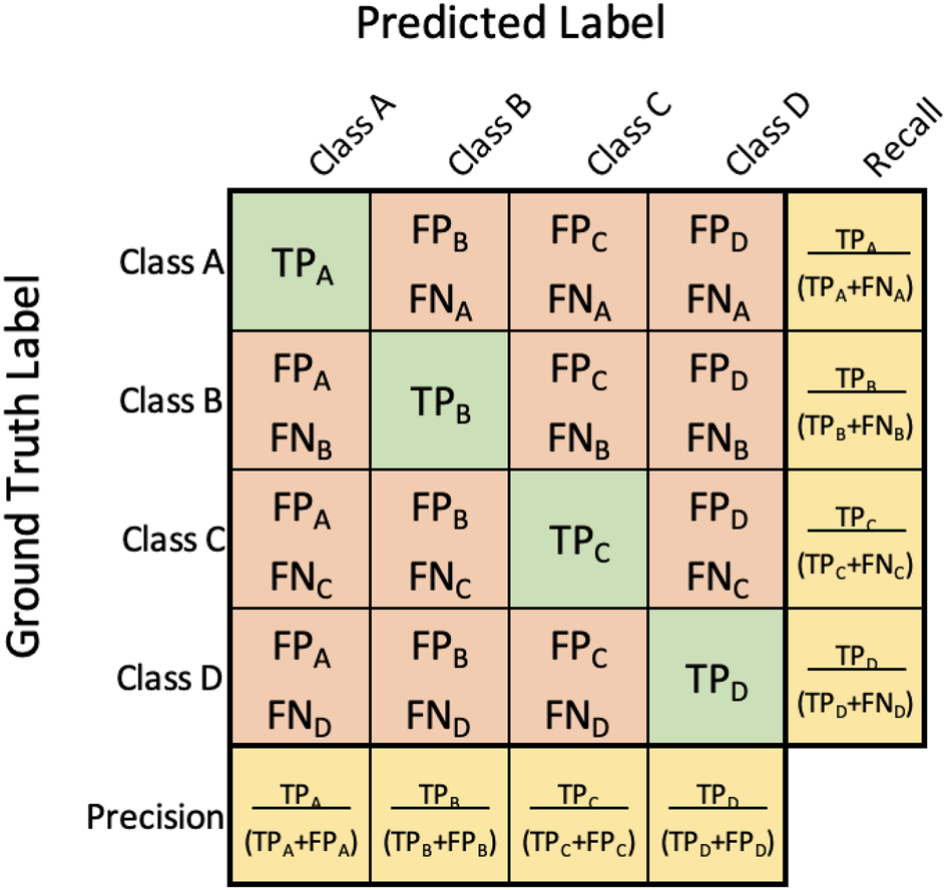
Confusion matrix template for a generic multi-class classification problem, showing how precision and recall are calculated for each class.

Another common tool used to measure the number (percentage) of correct and incorrect detections for each individual class based on the ground truth data is the confusion matrix. Figure 3 shows how a confusion matrix is defined for four generic classes. Rows represent the ground truth for each class and columns show the predicted results.

**Figure 4:**
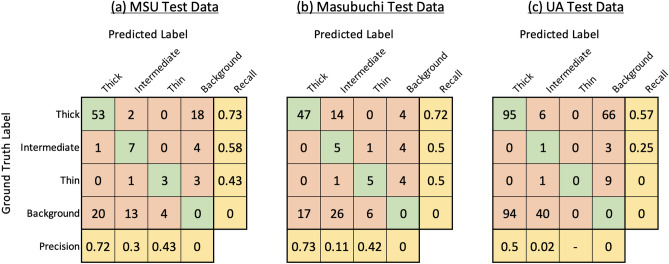
Confusion matrix showing results obtained by applying the proposed algorithm to all three test datasets (MSU, Masubuchi, and UA).

In our research, we have considered each flake within an image for evaluation. Following common practice in the field of object detection, we use an IoU of 0.5 to determine our numerical scores^32,46^ . Therefore we consider a true positive example to be when individual flake was detected and classified correctly considering the IoU. Incorrect detections occur when a flake is not detected at all (i.e. it is considered by the algorithm to be part of the background substrate), when it is detected as an incorrect class, or when the background substrate is detected as a flake. Figure 4a shows the confusion matrix showing the classification results on the 10 images in the test dataset. It can be seen that our trained model was able to achieve a sufficiently high number of correct detections, more notably for the Thin.

To assess the generalization of our method, we employed our trained algorithm using on ten images taken from two other datasets: The Masubuchi dataset and the UA dataset. We also compared our algorithm to the pre-trained algorithm published by Masubuchi et al.^32^.

To make a fair comparison between our method and Masubuchi method, we fed these unseen images from both datasets to our algorithm. Later we manually annotated the same images and labeled them as ground truth. Then we calculated the confusion matrix for both datasets based on the ground truths and detected results. The obtained confusion matrices when tested on 10 images from each dataset are shown in Fig. 4b,c.

**Figure 5:**
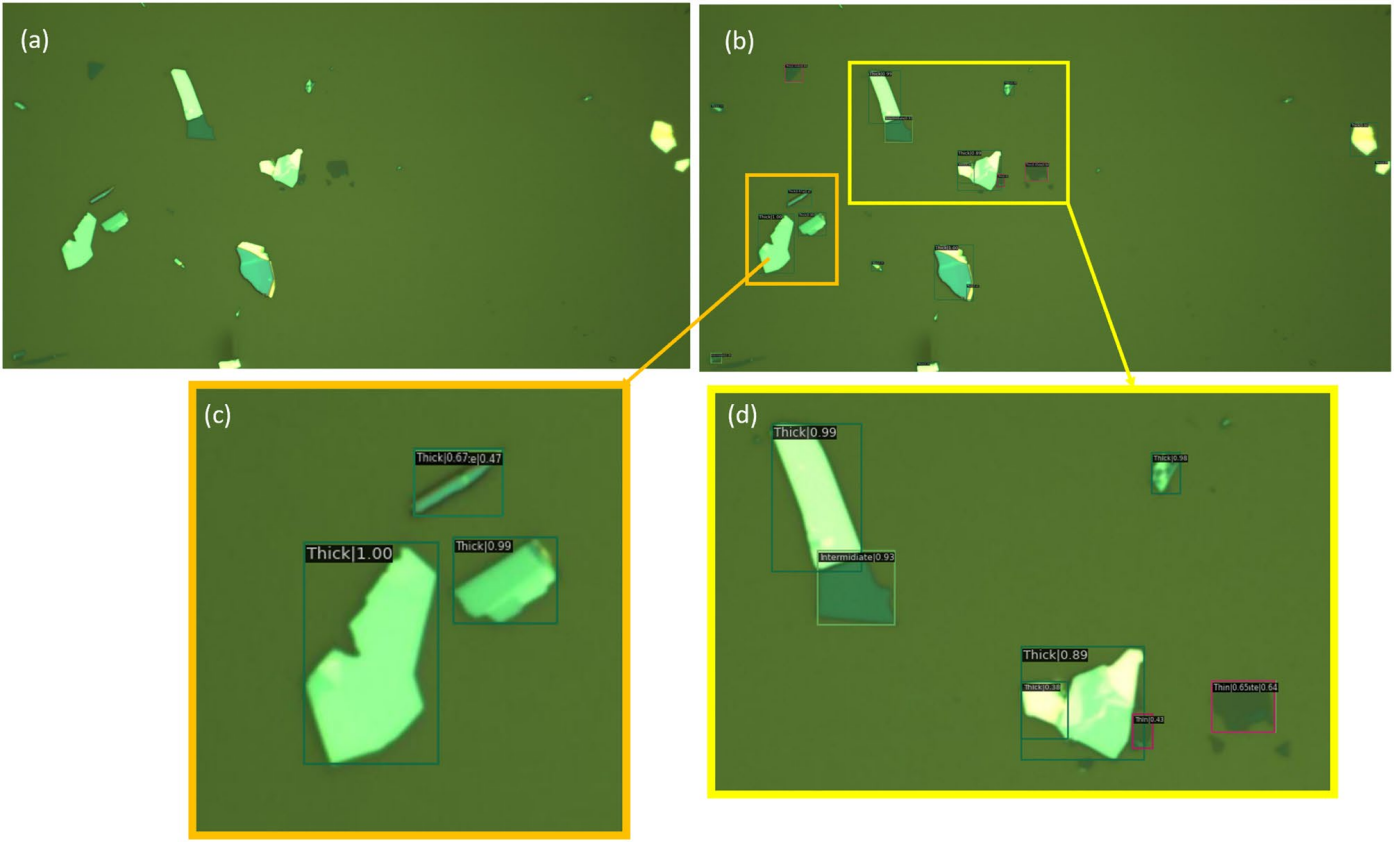
Results obtained from using the proposed algorithm on one image from the Masubuchi dataset; Part (**a**) shows the raw image, part (**b**) shows the labels generated by the algorithm, and parts (**c**) and (**d**) are zoomed-in regions showing the flake type and confidence score reported by the algorithm. We manually identified 16 flakes in the image, and the algorithm correctly classified 12 of them (microscope images obtained from https://doi.org/10.6084/m9.figshare.11881053 under the license CC BY 4.0).

**Figure 6:**
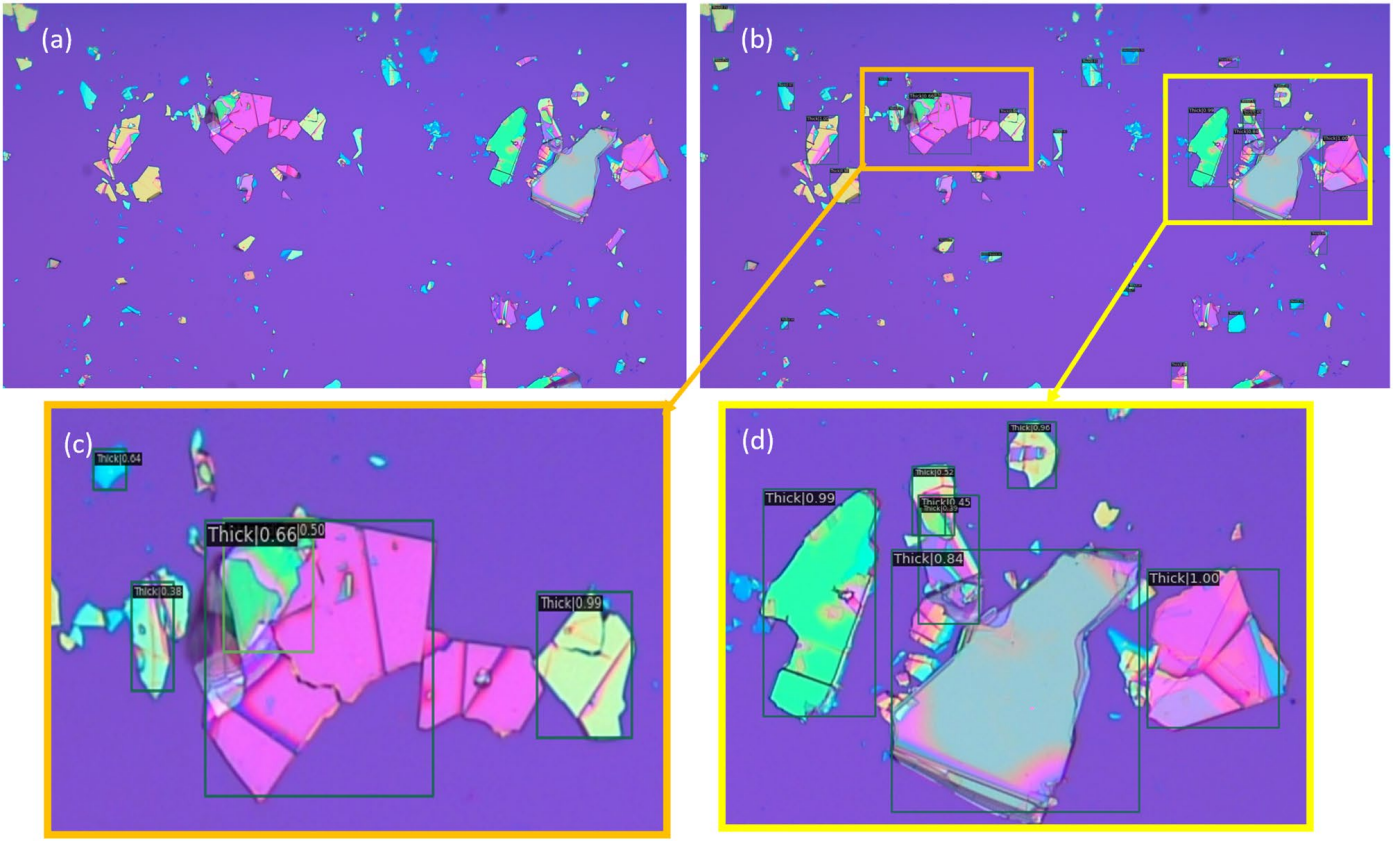
Results obtained from using the proposed algorithm on one image from the UA dataset; Part (**a**) shows the raw image, part (**b**) shows the labels generated by the algorithm, and parts (**c**) and (**d**) are zoomed-in regions showing the flake type and confidence score reported by the algorithm. We manually identified 32 flakes in the image, and the algorithm correctly classified 20 of them. (Microscope images obtained by Hugh Churchill and used with permission.).

Figures 5 and 6 show the detection results applying our proposed model to one image from the Masubuchi and UA datasets, respectively. In these images, our detector was able to identify 12 flakes correctly out of 16 annotated flakes within Masubuchi image, and 20 flakes correctly out of 32 annotated flakes for UA image.

## Discussion

**Figure 7:**
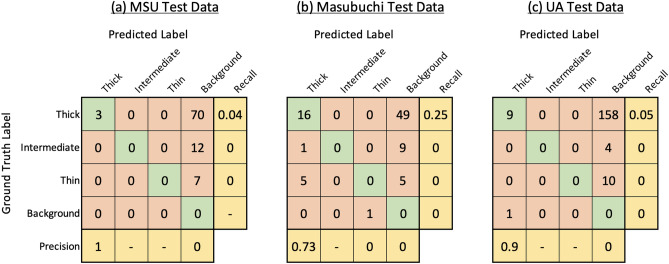
Confusion matrices showing results when applying Masubuchi’s pre-trained Mask R-CNN to our all three test datasets (MSU, Masubuchi, UA). The pre-trained model was unable to identify most manually labeled flakes in the test images.

Before training the DetectoRS model, we applied Masubuchi’s pre-trained Mask-RCNN on our own dataset. We discovered this pre-trained model was not able to perform well on our dataset. For many test images, including the image shown in Fig. 2a, the pre-trained model was unable to identify any flakes. For other images, the pre-trained RCNN was able to identify only a small subset of the total number of flakes. Complete results of using the Masubuchi method on all three dataset are presented as confusion matrices in Fig. 7. We note that one cause of the poor performance of their algorithm is that we modified the labeling procedure and criteria. Future work will explore a robust, standardized method for data labeling so that algorithm performance can be compared more directly.

In addition, future work will explore integrating the proposed algorithm with an optical microscope to make the detection pipeline fully automatic. We also intend to collect and label data associated with additional 2D materials, expanding beyond hBN.

## Data Availability

To help other researchers and scientists explore improved methods for solving 2D material object detection, the software and data used in this study is available on GitHub: http://github.com/BMW-lab-MSU/hBN_Detection. The software and data are published under the open-source BSD 3-Clause License. In addition, the repository has been archived on Zenodo and has received a digital object identifier (DOI): 10.5281/zenodo.7576917.
